# Research on lightweight algorithm for gangue detection based on improved Yolov5

**DOI:** 10.1038/s41598-024-57259-9

**Published:** 2024-03-20

**Authors:** Xinpeng Yuan, Zhibo Fu, Bowen Zhang, Zhengkun Xie, Rui Gan

**Affiliations:** School of Coal Engineering, Shanxi Datong University, Datong, 037000 China

**Keywords:** Yolov5s, Coal gangue recognition, EfficientVIT, Attention mechanism, Loss function, Computer science, Environmental sciences, Engineering

## Abstract

In order to solve the problems of slow detection speed, large number of parameters and large computational volume of deep learning based gangue target detection method, we propose an improved algorithm for gangue target detection based on Yolov5s. First, the lightweight network EfficientVIT is used as the backbone network to increase the target detection speed. Second, C3_Faster replaces the C3 part in the HEAD module, which reduces the model complexity. once again, the 20 × 20 feature map branch in the Neck region is deleted, which reduces the model complexity; thirdly, the CIOU loss function is replaced by the Mpdiou loss function. The introduction of the SE attention mechanism makes the model pay more attention to critical features to improve detection performance. Experimental results show that the improved model size of the coal gang detection algorithm reduces the compression by 77.8%, the number of parameters by 78.3% the computational cost is reduced by 77.8% and the number of frames is reduced by 30.6%, which can be used as a reference for intelligent coal gangue classification.

## Introduction

China's energy structure means that coal will remain the primary source of energy consumption for the foreseeable future^1^. Coal gangue is inevitably mixed in with coal during the mining process, and has become a major source of solid pollution that threatens the environment^2^. The classification and treatment of coal gangue have become an important topic due to increasing environmental awareness and the need to recycle resources. This is instrumental in promoting the clean utilization of coal. The mainstream of current gangue recognition technology development is the application of deep learning methods. These methods are compared to density recognition, hardness recognition, alternative methods for density and hardness, and alternative methods for grayscale and texture as discriminative features of ganglion recognition methods^3,4^.

Traditional gangue identification is a laborious and inefficient process. In recent years, several scholars have conducted extensive research on gang identification. Zhang and his team^5^ proposed a gangue sorting system based on the γ-ray dual-energy projection method^6^. This method utilizes two γ-ray sources with different energies as an excitation and accurately identifies gangue through the intensity of the radiation flux produced by its irradiation to the gangue. Yang and his team also proposed a method for gangue identification and sorting using dual-energy γ-rays^7^, which is based on the projection method. In their study, He et al.^8^ used X-ray projection to identify gangue. They set a threshold based on the energy attenuation of coal and gangue to X-rays and compared the energy attenuation of the target to X-rays with the threshold during detection to recognize gangue. The radiometric method-based gangue sorting systems have several drawbacks, such as high cost and long-term maintenance expenses. Additionally, the radiation generated by the radiometric detectors may pose a threat to the workers' health^9^. Vibration detection methods typically include mechanical sensors, electrical sensing, and hydraulic systems. While the vibration signal has the advantages of being easy to detect, having strong anti-interference ability, and being conveniently transmitted, it is not conducive to accurate mining and causes additional damage to the coal mining machine. Furthermore, it requires alteration of the position during the coal mining process, which affects equipment maintenance^10^. Vibration detection methods are utilised to differentiate between coal and rock by acquiring signals from the body of the coal mining machine or the top and bottom of the rock, and cutting the coal and rock seams at different frequencies and vibration amplitudes. Electromagnetic detection techniques such as radar detection, THz detection, and electron spin resonance methods are commonly employed. Traditional methods for detecting vibrations mainly consist of mechanical sensors, electrical sensing, and hydraulic systems. These methods rely on the differences in physical properties between coal and rock and utilize various properties of electromagnetic waves, such as velocity, time, phase, and return loss rate, as they propagate through coal and rock layers to accurately identify coal and rock. Radar detection has become a commonly used identification method due to its large detection range, high accuracy, and strong anti-jamming capability. Additionally, the THz sounding method analyses time delays and decay amplitudes to provide insight into data across different rock formations. The electron spin resonance method is a technique that uses electromagnetic waves emitted by coils and antennas to measure and evaluate the power of received signals. This method enables the detection of cracks, fissures, and other imperfections present in the interior of coal rock bodies, providing effective data for underground gas extraction in coal mines. While the electromagnetic detection method is versatile and easy to implement, it cannot be used on a large scale due to limitations in coal properties and the high number of interference factors affecting the signal. These factors cause quick signal attenuation, resulting in a significantly reduced accuracy rate^11,12^. Zhao et al.^13^ proposed a method for recognizing coal gangue using the CornerNet-Squeeze deep learning network model. This method effectively reduces conveyor belt background interference during target detection and has achieved positive results. Cao et al.^14^ also proposed a deep learning-based technique for recognizing gangue images. The Inception model is utilised, and transfer learning techniques are employed to share the weights and biases of the convolutional layers of the trained model for more efficient training and improved accuracy. Gao et al.^15^ proposed a lightweight coal gangue identification method based on the MobileNetV3-large module structure. The network model's performance was improved while maintaining a modest increase in the volume and complexity of the model parameters, resulting in increased accuracy compared to the original model. Lei et al.^16^ and others have improved Yolov3-M, resulting in faster convergence and improved recognition accuracy on small samples. Cai et al.^17^ have proposed a Yolov4-based deep learning network that enhances overall detection accuracy by modifying K-means initial anchor frame parameters. Zhang et al.^18^ proposed an enhanced Yolov5 model using the AdaBelief optimization algorithm, resulting in a 2.27% improvement in recognition accuracy. Chang et al.^19^ proposed a coal gangue recognition method using a Yolov5m improved model with DIOU-NMS recognition accuracy enhancement, resulting in improved tracing frame accuracy. Shenke et al.^20^ proposed an improved Yolov5s algorithm that enhances mean accuracy by 1.7% through linear scaling of anchor frames obtained by clustering the K-means algorithm. Gui et al.^21^ used the CBA-YOLO model to improve the average detection accuracy of gangue by 3.4% compared to the Yolov5 model. Although previous studies have improved the accuracy of identifying coal gangue to varying degrees, there are still some drawbacks. For instance, the number of model parameters increases, and the running time is longer. Using CIOU_Loss as an example, this method optimizes detection by computing the overlap region between the detection frame and the real frame. However, it is not effective when there is an inclusion phenomenon between the detection frame and the real frame. Additionally, the loss function converges slowly in the horizontal and vertical directions, which is insufficient for gangue classification.

Therefore, we made lightweight improvements to the Yolov5s model by replacing its backbone network with EfficientVIT. We also introduced C3_Faster in the HEAD module instead of the C3 module and removed the 20 × 20 feature map branch in the Neck region. These changes improved the convergence speed, reduced the number of parameters and computation of the model, and led to an increase in the detection rate. Mpdiou is used to achieve faster convergence and more accurate regression results, improving gangue differentiation.

## Yolov5s model

**Figure 1:**
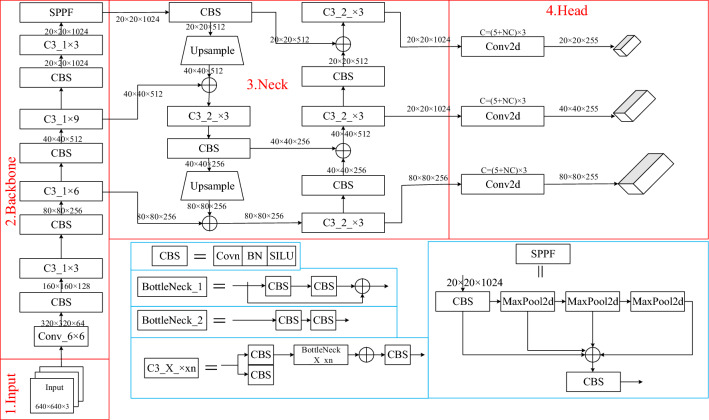
Structure of Yolov5s.

The Yolov5 object detection network is one of the most popular due to its accuracy and fast detection. Several different versions of the Yolov5 series have been proposed, such as Yolov5-t, Yolov5-s, Yolov5-m, Yolov5-l, etc. to target different inspection tasks, which makes the Yolov5 series more versatile for applications than the Yolov4 series. One of the most applied is Yolov5s, Yolov5s is the network with the shallowest network depth and the smallest width among the official 4 versions, Its network structure consists of four parts: the Input, the backbone, the Multiscale Feature Fusion network (Neck) and the Detection Head, while the Input adopts the Mosaic Data Enhancement and adaptive anchor frame computation and additional advanced techniques to enrich the dataset and obtain the best size anchor frame suitable for the dataset, the backbone network is mainly composed of CBS (Conv + BatchNorm + SiLU), C3, and SPPF modules, the Neck part adopts the Path Aggregation network (PANet), and the Head output is used for the output of the network prediction results. The structure of the Yolov5s is illustrated in Fig. 1.

## Improved Yolov5s model

**Figure 2:**
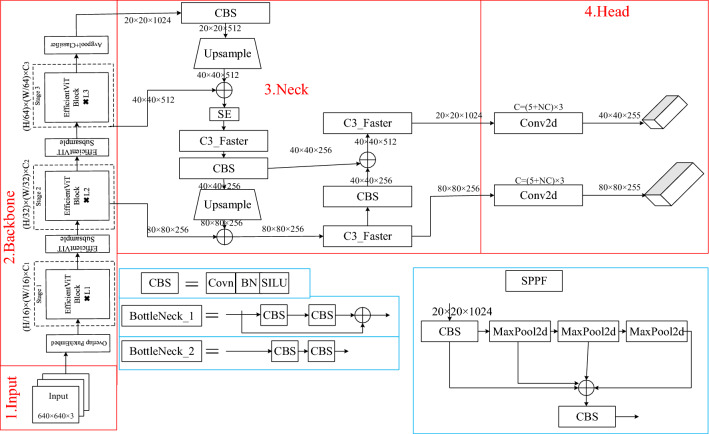
Structure of Yolov5s improved model.

The following improvements have been made to Yolov5s. The EfficientVIT network was proposed by Liu et al.^22^ to cascade groups of attentional modules and give different complete features to divide the attentional head, which saves computational costs and increases attentional diversity. Comprehensive experiments demonstrate that the efficiency is significantly better than existing effective models, yielding a better speed-capacity trade-off. Mpdiou is a modern bounding box similarity comparison metric based on minimum point distance, Mpdiou, proposed by Ma^23^ and others, which incorporates all the relevant factors considered in the existing loss functions, i.e., overlapping or non-overlapping areas, centroid distances, width and height biases while simplifying the computation process. C3_Faster, as a current Partial Convolution (PConv) technique proposed by Chen et al.^24^, performs spatial feature extraction more efficiently due to both reduced redundant computation and reduced memory access. Based on PConv, FasterNet, a novel family of neural networks, is additionally proposed, which achieves higher operation speed than others on different devices without compromising the accuracy of visual tasks. This is because the lightweight improvement of Yolov5s requires a reduction in both the number of parameters and the amount of computation, which can be achieved by all of the above methods and satisfies the experimental requirements. Thus, firstly, the entire backbone network in the original Yolov5s is replaced by the EfficientVIT network in the backbone module, secondly, the C3 module is replaced by C3_Faster in the HEAD module, and again, the Neck region of the Yolov5 model is appropriately streamlined, the 20 × 20 feature map branch, which has the largest sensory field and is suitable for detecting objects of larger size, is deleted, and finally Mpdiou is used to replace CIOU, while the SE attention mechanism is introduced, which is conducive to the model's better fusion of valuable features to improve the detection performance. A schematic of the structure of the improved model is shown in Fig. 2.

### EfficientVit

**Figure 3:**
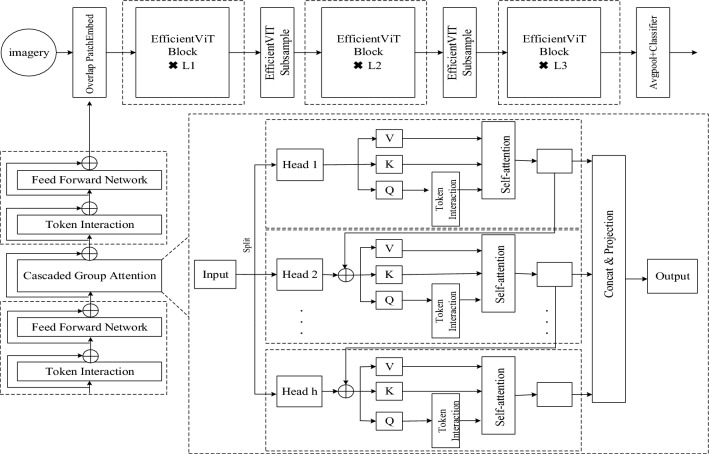
EfficientVIT structure.

EfficientVit is a lightweight network model. EfficientVit designs a different building block with a mezzanine layout, namely a single memoryless bound MHSA between valid FFN layers, which improves channel communication while increasing memory efficiency. EfficientVit also proposes a cascade group attention module that assigns different complete feature segmentations to the attention head^25^, and the overall framework is shown in Fig. 3. Containing three phases, each phase contains a number of sandwich structures, which consist of 2N DWConv (spatially localized communication) and FFN (channel communication) and cascaded packet attention. Cascading group attention differs from previous MHSA in that heads are first segmented and then Q, K, and V are generated. Alternatively, to learn richer feature maps and increase the model capacity, the output of each head is summed with the input of the next head. Finally, multiple header outputs are concatenated and mapped using a linear layer to obtain the final output, which is denoted as Eq:

1 $${X}_{ij} = Attn(X_{ij} W_{ij}^{Q} ,X_{ij} W_{ij}^{K} ,X_{ij} W_{ij}^{V} )$$

2 $${X}_{i + 1} = Concat[{X}_{ij} ]_{j = 1:h} W_{i}^{P}$$

3 $$X^{\prime}_{ij} = X_{ij} + {X}_{i(j - 1)} ,1 < j \le h$$

The jth head in Eqs. (1), (2) computes the self-attention on Xij, which is the jth partition of the input feature X_i_, i.e., Xi = [Xi1, Xi2, … , Xih] and 1 ≤ j ≤ h is the total number of heads, $W_{ij}^{Q}$, $W_{ij}^{K}$, and $W_{ij}^{V}$ are the projection layers that partition the input feature into different subspaces, and $W_{i}^{P}$ is a linear layer that projects the connected output features back to the input dimension that is consistent with the input.

Equation (3) where $X^{\prime}_{ij}$ is the sum of the jth input segmentation point X_ij_ and the (j-1)th head output $\widetilde{X}_{i(j - 1)}$ computed according to Eq. (1). It replaces X_ij_ as the original input feature for the j-th head when computing self-attention. In addition, another label interaction layer is applied after Q-projection, which allows self-attention to jointly capture local and global relations and greatly enhance the feature representation.

### Mpdiou improvement

The loss function is an influential component in neural networks whose main role is to measure the distance between the information predicted by the network and the desired information, i.e. The closer the two are to each other, the smaller the value of the loss function. The loss functions of the YOLO algorithm family mainly include the localization loss function (lossrect), the confidence prediction loss function (lossobj), and the category loss functions (loscls). The localization loss function used by Yolov5 is the CIOU function, which is computed as follows.

4 $$CIOU\_Loss = 1 - IOU + \frac{{\lambda^{2} (a,a^{gt} )}}{{c^{2} }} + \alpha \mu$$

5 $$\alpha = \frac{\mu }{(1 - IOU) + \mu }$$

6 $$\mu = \frac{4}{\pi }\left[ {(\arctan \frac{{w^{gt} }}{{h^{gt} }}) - \arctan \frac{w}{h}} \right]^{2}$$

Equations (4)–(6) in which *a* and *a*^*gt*^ are the centroids of the prediction and target frames, respectively, and *λ* is the Euclidean distance between the two centroids; *C* is the diagonal length of the smallest closed region of the predicted and target frames. *α* is the weight of the function; *μ* is the consistency of the aspect ratios of the two frames; Here, *h* and *w* are the height and width of the predicted frame, respectively. The *h*^*gt*^ and *w*^*gt*^ are the height and width of the target frames, respectively. The CIOU function mainly notices the overlapping parts of the prediction and target frames. The Mpdiou loss function is used.

Mpdiou is a bounding box similarity comparison metric based on the minimum point distance that includes all the relevant factors considered in existing loss functions. Mpdiou simplifies the similarity comparison between two bounding boxes and is suitable for overlapping or non-overlapping bounding box regression. Therefore, Mpdiou can be a decent alternative to the intersection and merging ratio as a metric for all performance metrics in 2D/3D computer vision tasks. It also simplifies the computation by directly minimizing the upper-left and lower-right point distances between the predicted bounding boxes and the actual labeled bounding boxes. Mpdiou is computed as follows.

7 $${\text{d}}_{1}^{2} = (x_{1}^{B} - x_{1}^{A} )^{2} + (y_{1}^{B} - y_{1}^{A} )^{2}$$

8 $${\text{d}}_{2}^{2} = (x_{2}^{B} - x_{2}^{A} )^{2} + (y_{2}^{B} - y_{2}^{A} )^{2}$$

9 $$M{\text{pdiou}} = \frac{A \cap B}{{A \cup B}} - \frac{{d_{1}^{2} }}{{w^{2} + h^{2} }} - \frac{{d_{2}^{2} }}{{w^{2} + h^{2} }}$$

In Eqs. (7)–(9) d_1_, d_2_ denote the intersection and minimum point distance, two arbitrary shapes: A, B ⊆ S ∈ Rn, and the width and height of the input image: w, h. Output: Mpdiou.Let $(x_{1}^{A} ,y_{1}^{A} )$, $(x_{2}^{A} ,y_{2}^{A} )$ denote the coordinates of the upper left and lower right points of A. Let $(x_{1}^{B} ,y_{1}^{B} )$, $(x_{2}^{B} ,y_{2}^{B} )$ denote the coordinates of the upper left and lower right points of B, respectively.

### C3-faster improvements

The object detection head is part of the feature pyramid used to perform object detection, which includes multiple convolutional, pooling, and fully connected layers, among others. In the Yolov5 model, the detection head module is mainly responsible for multiple object detection feature maps extracted from the backbone network. The module consists of three main parts. The C3 module is an essential part of the Yolov5 network and its main role is to increase the depth and receptive field of the network and improve the feature extraction capability. C3-Faster is implemented as C3-Faster by multiple Faster_Blocks, which can be used to replace the C3 module in Yolov5 thereby achieving accelerated network inference, where the Faster_Block is implemented by the lightweight convolutional PConv proposed in the literature^21^ in combination with additional operations. Replace the C3 module with C3-Faster in the HEAD module.

### Neck layer and prediction layer optimization

**Figure 4:**
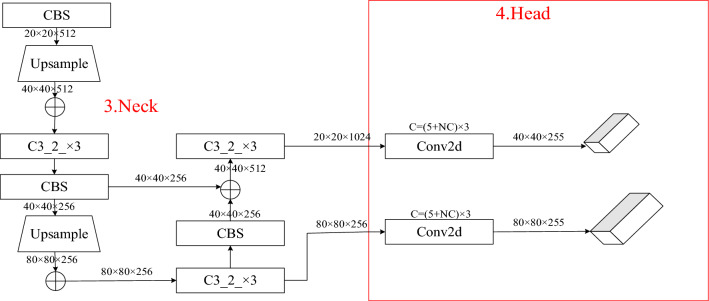
Improved neck and prediction structure.

The Neck region in the Yolov5 model uses a multipath structure to aggregate features and enhance network feature fusion. The size of the coal and gangue is too narrow with respect to the whole image, making the Neck region redundant for large object detection. In order to improve the model detection speed, the Neck region of the Yolov5 model is properly streamlined by removing the 20 × 20 feature map branch that has the largest receptive field and is suitable for detecting objects of larger sizes. Elimination is performed to reduce the model complexity and improve the real-time performance of detection. As shown in Fig. 4.

### SE attention mechanism

**Figure 5:**
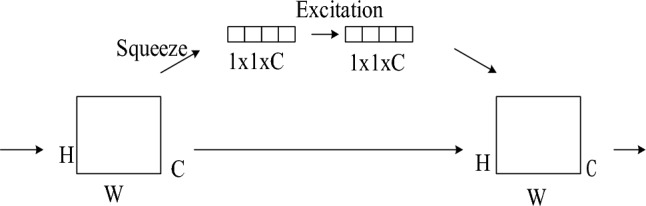
SE module diagram.

The SE attention mechanism is introduced into the original model to improve the object detection accuracy. The SE attention mechanism consists of three parts, namely, Tightening Squeeze, Incentive Expiration, and Feature Schema Calibration, with the main purpose of enhancing useful features. First, the global information of the feature maps is obtained by global average pooling, and the individual channels refine this information to derive the channel weights and adjust the weights of the original feature maps for better performance. The resulting feature maps are compressed along the spatial dimension, and the dimensionality of the feature maps is compressed using a global average pooling compression operation to turn each two-dimensional feature channel into a real number, with the output dimension matching the number of input feature channels. The feature map from W ∗ H ∗ C is compressed into a 1 ∗ 1 ∗ C vector by The feature map is compressed from W ∗ H ∗ C to a 1 ∗ 1 ∗ C vector by the Excitation operation using the completely connected layer acting on the feature map, and the Sigmoid activation function to obtain the normalized weights. The weight information is obtained through learning, and the weights are applied to the corresponding channels, and finally The scale operation is performed, and the weights of each feature channel obtained after the Excitation operation are multiplied with the original feature map channels one by one, and the generated feature vectors are multiplied with the corresponding channels of the feature map to obtain the weights of the corresponding channels, which are re-calibrated to the feature map. The SE module is shown in Fig. 5.

## Experiments and analysis of results

### Data acquisition and preprocessing

The experiments rely on independently collected coal and gangue datasets for training. Experimentally, in order to collect image data of coal and gangue, video recordings of coal and gangue under different illumination were recorded with black color as the background, and then frames were extracted from the video and the image of each frame was labeled by the labeling. Division of training and test samples. There are 738 images in the training sample, of which 500 are all coal and 238 are gangue, and 128 images in the test sample, of which 78 are coal and 50 are gangue, to complete the pre-processing process.

### Model training

**Figure 6:**
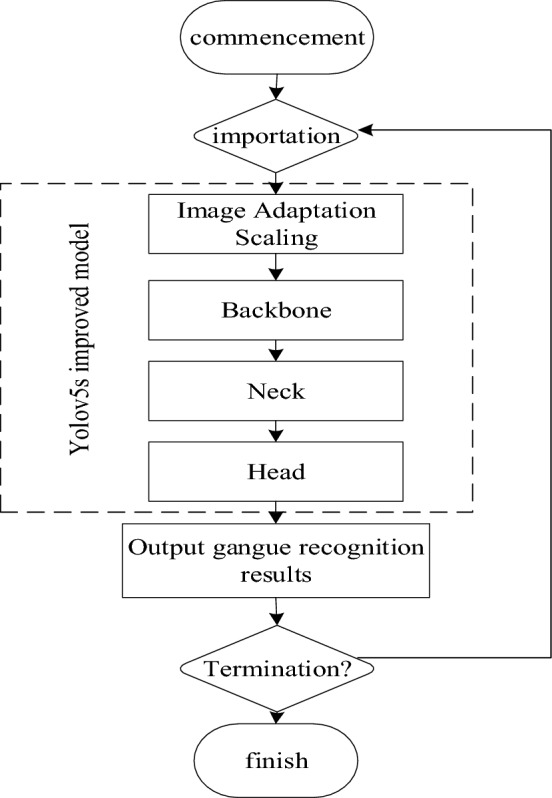
Yolov5s improved model gangue identification flow chart.

This experiment is based on the Pytorch 1.8.2 framework^26^, the operating system is Windows, the CPU is an Intel(R) Core(TM) i7-8750 with a CPU frequency of 2.2 GHz, the GPU is an NVIDIAGTX1050Ti, and the operating memory is 8 GB. An image of size 640 × 640 is input for training; Three channels are used as input to the image; The number of samples in the batch is 1; The weight decay parameter is set to 0.0005; The number of training rounds was chosen to be 100; The learning rate was chosen to be 0.01; and the evolutionary hyperparameter catch is set to false. The process of gangue recognition using the model with trained weights is shown in Fig. 6.

### Experimental results

Experiments were conducted to validate the gangue detection algorithm using Model, Params, FLOPs, FPS, and meanAveragePrecision (mAP) as performance metrics. The mAP is calculated as in Eq. (10).

10 $$mAP = \frac{1}{n}\sum\limits_{k = 1}^{k = n} {AP_{k} }$$

To test the performance of the modified algorithm, multiple sets of comparison experiments were performed by improving on the Yolov5s algorithm, as shown in Table 1.

**Table 1:** Comparison results of different improved models.

Model name	Params/M	FLOPs/G	Model/MB	mAP/%	FPS/(帧·s^−1^)
Yolov5s	70.1	15.8	14.4	94.9	37.5
Yolov5s-M^23^	70.1	15.8	14.4	94.9	38.9
Yolov5s-C^24^	57.8	12.6	11.9	94.6	39.3
Yolov5s-N	52.3	14.3	10.8	94.5	42.1
Yolov5s-E^22^	36.6	10.6	8.1	94.3	46.4
modified model 0	15.4	3.5	3.2	94.4	68.0
modified model 1	15.2	3.5	3.2	94.4	68.1

In the Yolov5s-M model, the CIOU loss function is replaced by. The FPS increases by 1.4 frames/s when the model size, number of parameters, number of computations, and mAP are kept constant, indicating that Mpdiou improves the model detection speed. The Yolov5s-C model is to replace the C3 part in the head module by C3_faster, which reduces the mAP by 0.3% with a reduction of 2.5 MB in model size, 12.3 M in parameter amount, 3.2G in computation amount, and 1.4 frames/s increase in FPS, which indicates that the use of the C3_Faster module improves the detection speed of the model and reduces model complexity. In the Yolov5s-N model, the 20 × 20 feature mAP branch is deleted from the Neck and Prediction layer. The model size was reduced by 3.6 MB, the number of parameters was reduced by 17.8 M, the amount of computation was reduced by 1.5G, the FPS was increased by 4.6 frames/s, and the mAP was reduced by 0.4%. The removal of 20 × 20 feature map branches can improve the detection speed and reduce the complexity of the model. The Yolov5s-E model replaces the backbone network in the Yolov5s network structure with the lightweight network EfficientVIT. With a reduction of 6.3 MB in the model size, 33.5 M in the amount of parameters, 5.2G in the amount of computation, and an increase of 8.9 frames/s in the FPS, the mAP decreases only by 0.6%, which indicates that using EfficientVIT as a network can improve the detection speed and reduce the model complexity of the model. Modified model 1 is based on modified model 0 with the addition of the SE attention mechanism. This addition allows the model to have fewer parameters, simplifying it further. Adding the SE attention mechanism to the improved Yolov5s resulted in only a 0.4% reduction in mAP, an 11.2 MB reduction in model size, a 54.9 M reduction in parameters, a 12.3G reduction in computation, and a 30.6 fps increase in FPS. Comparison results show that the improved Yolov5s model is able to significantly reduce the model size, number of parameters, and computation while sacrificing only 0.5% detection accuracy. The main purpose of this paper is a lightweight improvement, hence the modified Yolov5s is identified as the final optimized model.

**Figure 7:**
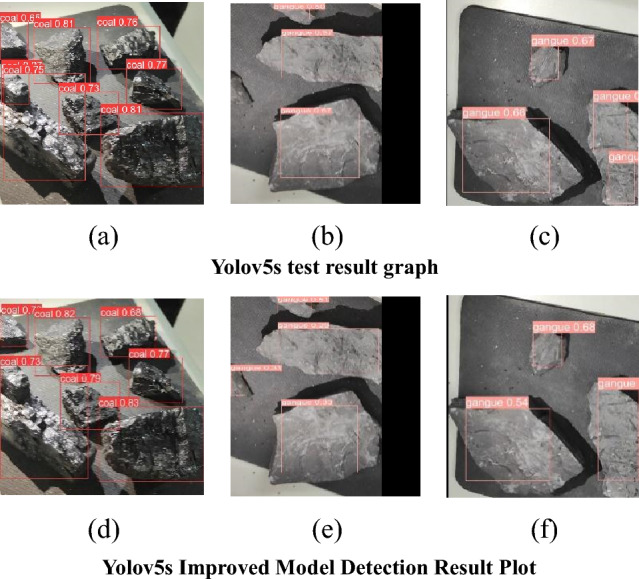
Comparison of detection effect.

To further test the detection effect of the modified Yolov5s coal gangue detection model, the detection results of the two algorithms of Yolov5s, the modified Yolov5s for coal gangue, are compared; see Fig. 7. The red color indicates coal and the pink color indicates gangue. In the detection comparison figure, a, b, and c are the detection results of the Yolov5s algorithm, and d, e, and f are the detection results of the Yolov5 improved model. Both the Yolov5s algorithm and the modified Yolov5s algorithm accurately detect coal tar, but the modified Yolov5s algorithm has better overall detection results. The Mpdiou loss function used enables better detection of detection frames, increases receptive field, and improves image feature extraction, e.g., a and d compare the detection frames of d for full scan detection. The modified Yolov5s algorithm in the b and e comparison plots solves the leak detection problem in the Yolov5s algorithm. The modified Yolov5s algorithm in the comparison plots of c and f solves the overlapping detection frame problem in the Yolov5s algorithm. Among them, the Yolov5s algorithm detects gangsters with up to 95% accuracy, while the modified Yolov5s algorithm detects gangsters with up to 93% accuracy. Compared to the modified Yolov5s, there is a 2% decrease in accuracy between the two, but the experimental results show that the modified Yolov5s are superior in terms of detection effectiveness. In summary, the modified Yolov5s algorithm performs better than Yolov5s for detection.

### Comparison of results of different algorithmic models

We compared the results of the different algorithmic models using different versions of the detection model (including Yolov3, Yolov4, Yolov5, Yolov6, Yolov7 and Yolov8), and the results of the comparison are shown in Table 2. The models' hyper-parameters and training parameters are set to their default values. Yolov3 and Yolov6 are executed using the source code that accompanies yolov8, while Yolov4 and Yolov7 use its official source code. The benefits of the Yolov5s model are evident. Although the accuracy of the improved model is reduced, the model size, number of parameters, and computation are significantly streamlined. As a result, the improved model can detect gangue in less time, demonstrating the advantages of the proposed model.

**Table 2:** Comparison of results of different algorithms.

Model name	Params/10^6^	FLOPs/G	Model/MB	mAP/%
Yolov3-tiny	12.1	19.0	24.3	91.1
Yolov4s	64	29.9	244	77.13
Yolov6s	16.2	44.0	32.8	92.1
Yolov7-tiny	6.0	13.2	12.3	90.8
Yolov8s	11.1	28.4	22.5	91.3
Yolov5s	70.1	15.8	14.4	94.9
Modified model 1	15.2	3.5	3.2	94.4

## Conclusion

The Yolov5s model utilises EfficientVIT as the backbone network and C3_Faster to replace the C3 component of the head module. This results in a lightweight model with improved detection speed. Additionally, the 20 × 20 feature map branch in the Neck region is removed to reduce model complexity. The SE attention mechanism is also introduced with the Mpdiou loss function to enhance attention on essential features of the model.

Compared to the Yolov5s algorithm, this algorithm reduces the model size, number of parameters, and amount of computation by 77.8%, while improving the FPS by 30.6 frames/s. However, there is a sacrifice of 0.5% average accuracy. The results indicate that the enhanced coal gangway detection model exhibits a significant reduction in model complexity. However, the average detection accuracy is slightly lower than that of the Yolov5s algorithm. Future work could focus on improving the accuracy of the model.

## Data Availability

The datasets generated during and/or analysed during the current study are available from the corresponding author on reasonable request.
